# A systematic review of global COVID-19 vaccine PPPs: drivers and barriers to governance alignment

**DOI:** 10.3389/fpubh.2025.1727808

**Published:** 2025-12-01

**Authors:** Xuejing Qi

**Affiliations:** Department of Public Governance and Management, Ghent University, Gent, Belgium

**Keywords:** COVID-19, COVID-19 vaccine, public-private partnerships, global health governance, vaccine supply chain, policy coordination

## Abstract

**Background:**

Public-private partnerships (PPPs) have emerged as a prominent governance model for vaccine equity in the COVID-19 vaccine supply chain. Previous studies focus on evaluating PPP’s performance, lacking multi-dimensional analysis on the drivers and barriers that shape public and private actors’ willingness to participate in PPPs.

**Method:**

Following the PRISMA 2020 guidelines, a systematic review using the Web of Science (WoS) database was conducted to identify empirical factors influencing stakeholders’ preference for PPPs and the alignment between sectors was explored by qualitative content analysis.

**Results:**

Three main categories of private sector drivers were identified, including regulatory facilitation, financial incentives and reputational incentives. While four sets of barriers emerged, including the political environment, economic and logistic constraints, and the contractual obligations. For the public sector, motivations centered on ethical considerations, national interest protection, and institutional advantages, while participation was also constrained by vaccine nationalism and administrative lag. The analysis demonstrates the degree of alignment and misalignment among these governance factors.

**Conclusion:**

Based on the analysis of factors, this study proposes the Governance Alignment Framework (GAF) as a conceptual tool to pair the profits of different sectors and guide governments and public sectors to improve the developmental-steering capacities to better align private incentives with public value during the pandemic.

## 1 Introduction

Since the outbreak of the COVID-19 pandemic, health policy practitioners and relevant administrative bodies have been encouraged to engage stakeholders from diverse sectors in the COVID-19 vaccine supply chain because traditional approaches have limitations in improving and securing a durable vaccine supply during the urgent situation. The magnitude of this challenge needs the collaboration between public and private entities. In this study we specifically focus on PPPs that are defined as cooperative arrangements based on mutual commitment between a public sector organization and any external organization beyond the public sector (1).

The integrated-care literature, widely cited typologies clarify what is being integrated and at which level, thereby illuminating governance of cooperation. For example, Valentijn’s (2) “Rainbow” model links micro- (clinical/individual), meso- (professional/organizational), and macro-level (system/policy) integration. Busetto et al. (3) propose a taxonomy of “what to integrate,” covering clinical, professional, organizational, and system dimensions to align design and evaluation. Goodwin (4) stresses that co-governance and integration are complex, context-dependent processes rather than single interventions. Taken together, these insights motivate a focus on governance alignment in the vaccine-supply-chain context.

The application of PPPs has a rich history in the field of public health and vaccines. Notable early examples include the establishment of the International AIDS Vaccine Initiative (IAVI) in 1996, the Global Alliance for TB Drug Development in 2000, and the Medicines for Malaria Venture (MMV) in 1999. These partnerships were dedicated to addressing neglected diseases through drug and vaccine development (5), with venture-capital investment serving as their main model. PPPs have also played an essential role in vaccine procurement and distribution. For example, Biovac Institute (BI), formed as a PPP in 2003, has actively engaged in vaccine research, manufacturing, and supply. Between 2004 and 2014, BI successfully fulfilled its responsibilities by procuring and distributing vaccines at globally competitive prices (6), contributing to immunization outcomes in South Africa. Other notable examples include the DREAMS partnership focused on HIV/AIDS relief for women and girls, as well as the Go Further program launched in 2018 that targets AIDS and cervical cancer. Drawing from these past experiences provides valuable insights into why PPPs should be leveraged as a key approach for tackling pandemics while enhancing global COVID-19 vaccine supply chain efficiency.

Although PPPs have garnered substantial attention from researchers during the pandemic, most of their focus has been on analyzing these partnerships from a pragmatic standpoint, investigating PPP projects, and the stakeholders and participants. For example, Holzer et.al (7) focus on the PPP- COVAX and emphasize its role for low- and middle-income countries, Fajber (8) demonstrates the relationship between the human rights and PPP’s objectives and von Achenbach (9) examines COVAX from the public-law perspective. However, only a limited number of systematic reviews have explored the positive and negative factors that stakeholders face when participating in such partnerships - factors that are crucial for the success of PPPs. WHO’s Integrated People-centered Health Services (IPCHS) framework principles articulate continuity, coordinated governance, and accountability across settings (10). This motivates a shift from listing sectors to clarifying how integration is actually designed into PPPs, for who decides, with which instruments and under what incentives. Therefore, this article aims to address this gap by conducting a comprehensive systematic review of existing literature on PPPs and empirical evidence to answer the following research questions:

*RQ1*: “What are the motivations for the public/private sectors to opt for PPPs in COVID-19 vaccine supply chains?”

*RQ2*: “What barriers hinder the public/private sectors from embracing PPPs in COVID-19 vaccine supply chains?”

Building upon these questions, this article makes both empirical and theoretical contributions to the improvement of PPP design and coordination. Empirically, a summary of public and private partners’ motivations is provided for engaging in PPPs, differentiating them into positive and negative dimensions. Beyond identifying motivations, we reframe PPP engagement as a public–private interest-alignment problem among governance elements, rather than a project performance appraisal. Theoretically, we develop a novel Governance Alignment Framework, which integrates insights from four complementary theories, including Public Value (11), Resource-Dependence (12), Legitimacy/Stakeholder (13), and Public Goods theories (14) to show how institutional mechanisms make public and private objectives compatible.

## 2 Methods

**Figure 1:**
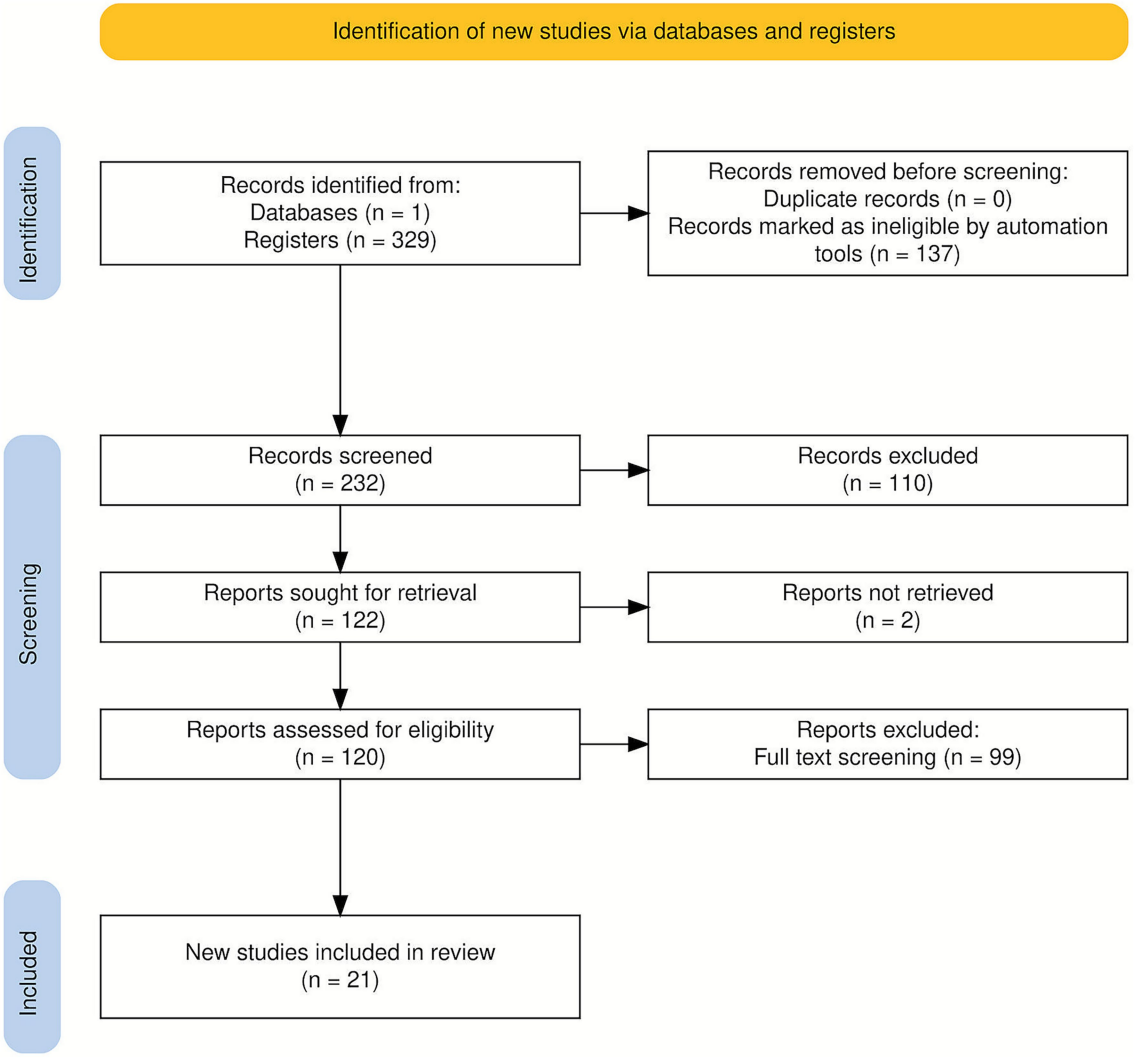
Preferred reporting items for systematic reviews and meta-analyses (PRISMA 2020) flow diagram (53).

This review was conducted in accordance with the PRISMA 2020 guidelines (15). A PRISMA flow diagram (Figure 1) illustrates the process of literature identification, screening, and inclusion, along with the rationale and inclusion criteria.

### 2.1 Search strategy

The research strategy followed the PRISMA 2020 recommendations. Initially, we conducted an electronic search in the ISI Web of Science database using topic-related keywords to ensure consistency and relevance because our research focus lies not in clinical trials, drug efficacy or epidemiological studies, but rather in the fields of policy science and public administration. ISI Web of Science provides comprehensive coverage of high-quality, high-impact Social Sciences Citation Index (SSCI) and Arts & Humanities Citation Index (A&HCI) journals. These publications serve as core platforms for disciplines such as public administration, political science, organizational theory, and global governance, precisely encompassing the theoretical foundations and policy analysis literature required for our proposed framework.

Gray literature, reports, and preprints were excluded to ensure methodological consistency, as they have obvious preference.

Second, given our focus, results were refined to include peer-reviewed journal articles in English and categorized under relevant subject areas. To ensure transparency and replicability, only open-access articles were retained. The search covered the period January 2020 to September 2025, with the final update conducted on September 1, 2025.

### 2.2 Inclusion and exclusion criteria

Two steps were followed to select articles for inclusion in the final analysis for coding.

First, titles and abstracts were screened to exclude articles that (i) focused primarily on vaccine research in clinical development; (ii) provided only general discussion on the role or significance of PPPs; (iii) presented purely conceptual papers about their potential development in the future.

Second, full-text screening was conducted to include articles that met the following criteria:(i) “The study examined PPPs within the context of the COVID-19 vaccine supply chain.”; (ii) “An empirical or descriptive approach was utilized to examine PPPs implementation in the COVID-19 vaccine supply chain.”; (iii) “Discussion regarding drivers and barriers of stakeholders’ participation in PPPs was provided.”; (iv) “PPPs did not concern the health status of a specific demographic, and deliberations were conducted at least at the national or multinational level.

Duplicate removal was deemed unnecessary because we searched a single database.

The analysis focused on understanding the drivers and barriers shaping public and private sectors’ decisions to adopt PPPs within the COVID-19 vaccine supply chain, without limiting it to a specific stage. While PPPs have been extensively discussed in vaccine research and development, equal attention should be given to distribution and management aspects. The qualitative coding procedure followed conventional content analysis (16). See Supplementary Table S1 for the full WoS TS query and Supplementary Table S2 for the full-text eligibility checklist summary.

### 2.3 Data extraction and analysis

Coding categories were designed based on the research questions. Two main coding categories were developed to organize and interpret the literature.

(1) Field synthesis attributes: to better analyze researchers’ focus on the PPPs in the vaccine supply chain during the pandemic, we developed a set of attributes summarizing the key dimensions of the field, including the level of focus, the critical PPPs case analyzed, focused research questions and designs, the stage of vaccine supply chain studied, and the main actor focused.
(2) Conceptual attributes: to answer two research questions, we extracted and categorized following attributes: drivers and barriers influencing the participation in PPPs in COVID-19 vaccine supply chain for public and private sectors.

To ensure coding uniformity, the first author independently coded the full corpus. After a two-week washout, the author conducted an intra-rater stability audit on a random 20% subsample. To further strengthen credibility, we implemented a blind double-coding of a random 20% subsample of coded text units (segments) across all 21 studies (*n* = 39 of 195) by an independent peer, followed by consensus adjudication and codebook refinement.

For boundary cases, for example, distinguishing vaccine nationalism from protection of national interests, we applied pre-specified decision rules anchored in the study’s analytic baseline and focus.

Given the multi-label structure and unbalanced prevalence of several codes, we report Krippendorff’s *α* (nominal) as the primary reliability indicator because it accommodates multi-label coding and sparse cells. Krippendorff’s α = 0.87, indicating strong reliability (≥0.80 is commonly considered sufficient for drawing conclusions).

### 2.4 Limitations and potential bias

This review follows PRISMA 2020 guidelines, but it has some limitations and selection bias that should be acknowledged.

Only articles in English were included, so it is possible that some important analyses related to the topic were not captured. The single database selection although ensures consistency in peer-review standards but narrows coverage and may shape the evidence profile reported below. We did not include preprints, books, conference proceedings, policy briefs, or other gray literature, so the distributions we report should be interpreted as patterns in the published record, not as the underlying prevalence of PPP practices across settings or stages.

## 3 Results

### 3.1 Study selection

A total of 120 records were retrieved from the Web of Science database. After removing irrelevant records and applying the inclusion and exclusion criteria, 99 articles were retained for full-text assessment. Following the second-stage screening, 21 studies met all eligibility requirements and were included in the final synthesis. The selection process, conducted in accordance with the PRISMA 2020 guidelines (15), is depicted in Figure 1.

### 3.2 Field synthesis

#### 3.2.1 Geographical focus

Analyzing the scope of public and private cooperation reveals a highly uneven landscape. The global level is the most frequently studied that there are 13 studies (61.9%). A small number of studies have researched the national level (6 studies; 28.6%), including four on the United States (19.1%), one on the United Kingdom (4.8%), and one on Canada (4.8%). Two additional studies (9.52%) examine PPPs at the regional level, which are relevant to the EU and Africa, respectively. It is worth noting that no studies directly examine PPP cases in low- and middle-income countries (LMICs), although a substantial share of global-level studies focuses on vaccine inequity experienced by lower-income economies. This pattern reflects the concentration of research on PPPs in higher-income economies (HICs), where institutional capacity and resources facilitate such collaborations. For example, Kim et al. (17) analyze the U. S. Federal Retail Pharmacy Program and its cooperative model between government and pharmacy providers, highlighting operational challenges faced by pharmacies. Lexchin (18) investigates the attitude of Canadian government to protecting intellectual property rights of enterprises within the partnership. Wolff and Ladi (19) discuss how the “Team Europe” initiative strengthened global health PPPs and promoted coordinated Europeanization.

The prominence of global and high-income settings is consistently reported alongside data accessibility, institutional transparency, and documentation density. Multilateral platforms and HIC agencies produce public contracts, guidance, and performance reports that are more readily citable than LMIC operational records. This publication ecology raises the observation probability of global and HIC cases in peer-reviewed sources.

#### 3.2.2 Stages of the vaccine supply chain

Examining the stages of the COVID-19 vaccine supply chain reveals that PPPs have been most frequently applied in the distribution phase (12 studies; 57.1%), followed by research and development (3 studies; 14.3%). Several studies covered multiple stages, such as distribution and vaccination (2 studies; 9.5%), research and distribution (3 studies; 14.3%), and research and production (1 study; 4.8%). Although the stages and levels differ, the literature consistently demonstrates that PPPs possess institutional advantages in achieving collaborative objectives and attracting diverse stakeholders. At the global level, for example, the COVAX functions as a global pooling mechanism to pool demand and de-risk development and procurement (8). Dose-sharing through COVAX has been described as a win-win scenario for both governments and pharmaceutical companies (8).

The distribution stage was the longest-running in the COVID-19 vaccine supply chain, beyond the initial R&D and early manufacturing, so it accumulates more observable events over time. It also generates abundant, standardized documentation compared to other stages. Another reason is that the distribution process directly concerns vaccine access, a core objective of many PPPs.

Having established the main areas and stages of research focus, the analysis now turns to the actors examined in the reviewed studies. Among 21 articles, six (28.6%) analyze the roles and perspectives of non-profit organizations, government, and other public sectors. 13 articles (61.9%) focus on both public and private sectors, while exclusive analysis of private-sector actors is least represented, with only two studies (9.5%) that focused on pharmaceutical enterprises. Regarding specific PPP cases, more than half of studies (13 instances, 61.9%) focus on COVAX. This reveals a strong interest in the unique characteristics of COVAX itself. For example, Fawole et al. (20) introduce the insurance mechanism of COVAX, which would incentivize these wealthier nations to participate. Von Achenbach (9) analyses COVAX’s purchasing community, and its common investment and procurement mechanisms designed to provide a “fallback” for participating countries. Three studies explore cross-cutting cooperative mechanisms rather than individual cases. For example, Choi et al. (21) systematically introduce the use of adaptive contracts during the pandemic as a flexible tool to incentivize private-sector engagement. Furthermore, only one article (22) specifically investigates rare empirical perspective based on expert interviews, analyzing the motivations and constraints shaping private-sector participation.

These findings indicate that existing scholarship has concentrated heavily on COVAX and government-led partnerships, but research on private-sector perspectives and cross-case comparative mechanisms remains limited. This imbalance suggests an opportunity for future studies to explore the diverse incentives and governance structures that shape PPP participation beyond the COVAX model.

### 3.3 Conceptual synthesis (drivers and barriers)

#### 3.3.1 Drivers for private sector participation in the PPP

The reviewed articles identify multiple factors motivating private-sector actors to engage in PPPs during the COVID-19 pandemic. Three major themes emerge from the literature: regulatory and operational facilitation, reputational drivers, and financial benefits.

(a) Regulatory and operational facilitation

Private-sector participation was strongly influenced by supporting regulatory frameworks and operational assistance provided by PPPs.

*Simplifying administration and coordinating logistics*

COVAX, as the largest global alliance, centralized logistical responsibilities and distribution networks, reducing the operational burden on manufacturers (22). For instance, the HOPE Consortium (23) supplemented COVAX’s cold-chain capacity, addressing the unique logistical challenges of mRNA vaccines that require ultra-cold storage. In the U. S., the Centers for Disease Control and Prevention (CDC)–McKesson partnership enabled coordinated vaccine delivery across jurisdictions.

*Streamlining regulatory compliance*

Operation Warp Speed (OWS) in the U. S. provided companies with accelerated Food and Drug Administration (FDA) review and trial coordination (24). Similarly, the Coalition for Epidemic Preparedness Innovations (CEPI) serves as a collaborative platform uniting diverse partners toward a shared goal of vaccine development, while national regulatory authorities continue to apply their respective approval standards. COVAX provides companies with a unified pathway to access global markets without concerns about varying regulations across different countries (22). Meanwhile, it simplifies both registration procedures and associated costs.

*Ensuring legal and liability protection*

Through its No-Fault Compensation Program, COVAX offered a first-of-its-kind, international mechanism to compensate serious adverse events in AMC-eligible economies, complementing country-level indemnification and liability arrangements (25, 26). The Global Alliance for Vaccines and Immunization (GAVI) explicitly communicated to participating governments that manufacturers would receive immunity from legal claims related to vaccine side effects, a policy echoed by AstraZeneca (27).

(b) Reputational drivers

Private-sector involvement was also driven by reputational and ethical incentives.

*Shifting moral responsibility*

A pronounced disparity in vaccination rates exists across countries and populations. In the context of bilateral agreements, companies possess considerable pricing power and autonomy in selecting commercial partners. Conversely, when participating in COVAX, enterprises can adhere to the WHO-established vaccine allocation scheme, thereby deferring decisions regarding inequality to an external entity. During the distribution of COVID-19 vaccines, numerous conflicts have arisen concerning prioritization among populations, countries, and different demographic groups (25).

*Building socially responsible image*

Moreover, many companies sought to strengthen their ethical and socially responsible image. Corporate support for global vaccination was viewed as a visible contribution to public good. According to von Achenbach (9), ensuring access to vaccination is not only an ethical imperative but a public law obligation, reinforcing the link between corporate responsibility and public legitimacy.

*Responding to external pressure*

Investors, media, and international organizations urged companies to “do the right thing” by engaging in global health governance (22). The ICESCR reaffirmed corporate human rights obligations, while international initiatives like the COVID-19 Technology Access Pool (C-TAP) encouraged intellectual property sharing to accelerate the pandemic response (24).

(c) Financial benefits

Financial incentives remain a core motivation for private-sector involvement.

*Implementing substantial financing mechanisms*

PPP frameworks such as COVAX, CEPI, and OWS provided substantial push–pull financing mechanisms, including advance purchase agreements, subsidies, and pre-funding (21, 22, 28, 29). These mechanisms mitigated firms’ R&D risks and ensured financial returns even in the event of vaccine failure. For example, Moderna received over USD 10 billion in U. S. public investment through BARDA and OWS, without repayment obligations if development failed (24). Forman et al. (28) introduce a ‘sales’ tax credit for COVID-19 vaccines provided by the US government to companies as a type of subsidization as well.

*Expanding market access*

After initial high-income markets were saturated, COVAX provided an additional global channel for distribution. Moderna is a notable example of this scenario. The PPP initiative offered smaller companies with limited vaccine experience the opportunity to access the global market. PPPs granted firms entry to global procurement channels, maintained strong ties with powerful government and expanded the market area, which were aligned with long-term strategy for companies (20). The study also notes that Pfizer captured a significant portion of the US market, leaving little incentive for supporting other enterprises. Through the Coalition for Epidemic Preparedness Innovations (CEPI), vaccine-producing companies gained access to a streamlined procurement process. Initially designed as a global procurement mechanism (30, 31), COVAX establishes an efficient marketplace that can swiftly respond to market demands and effectively mobilize all available resources. Private enterprises participate in the COVAX platform, engaging in direct negotiations rather than seeking potential collaboration partners. The platform thus facilitated efficient matching between buyers and manufacturers, promoting timely and successful vaccine transactions.

*Incentivizing production*

AstraZeneca’s early production in mid-2020, supported by CEPI, exemplifies the success of this strategy (32). Early commitments play a crucial role in shaping a company’s development strategy. Financial mechanisms also promoted resource sharing in technology and infrastructure, thereby reducing costs and innovation risks (33).

In summary, PPPs provided the private sector with a combination of financial security, market expansion, and risk mitigation, reinforcing long-term incentives for engagement in global health cooperation.

#### 3.3.2 Barriers to private sector participation in the PPP

Despite the incentives discussed earlier, several factors have constrained private-sector engagement in PPPs during the COVID-19 pandemic. The identified barriers can be grouped into four interrelated domains: political environment, economic constraints, logistical and distribution challenges, and contractual limitations. Together, these challenges illustrate the complex political, economic, and institutional factors that limited private-sector engagement in PPPs.

(a) Political environment

Political dynamics significantly influenced the degree of private-sector participation in PPPs. During the early vaccine rollout, vaccine nationalism led many high-income countries to prioritize domestic access through bilateral agreements, leaving limited supply for global mechanisms such as COVAX (8). By the time multilateral PPPs were ready to operate, production capacity was already committed, reducing firms’ willingness to divert doses toward collective platforms.

In addition, vaccine diplomacy serves as a means for countries to bolster their soft power and secure geopolitical benefits, thus influencing the decisions of private companies. Initially, the term “vaccine diplomacy” referred to the vaccine donation policies of Russia, China, and India, but it has since been adopted by the United States and the European Union (25, 30). De Bengy Puyvallée and Storeng (25) associated vaccine diplomacy specifically to China, noting that all Chinese vaccines were distributed through bilateral channels. Sung et al. (26) detailed the percentages of vaccines in COVAX supply agreements, revealing that Sinovac had the lowest rate of supplying its products via COVAX. National policies impact corporate cooperation trends. A notable example is India’s imposition of export bans on vaccines and related materials, compelling the private sector to prioritize domestic markets.

Overall, these political pressures fostered a form of macro-level misalignment, where national interests and geopolitical ambitions outweighed collective public health objectives, discouraging firms from committing to multilateral PPP arrangements.

(b) Economic constraints

Some leaders of pharmaceutical companies exhibit positive attitudes toward PPPs, but practical implementation remains constrained by a lack of experience in cooperating with multilateral organizations (22). Although these leaders often express a strong interest in participating in PPPs, the challenge of aligning business interests with PPP objectives can lead to hesitation in making final decisions. On the other hand, private enterprises face skepticism from other participants due to their primary focus on profit. Indeed, from the opportunity cost perspective, vaccine manufacturers stand to gain more from direct negotiations with wealthy nations compared to PPPs, especially when the global vaccine supply is limited (22), which further intensifies corporate reluctance. The access to vaccines suggests a market-oriented approach (8) which has intensified commercial competition and diminished corporate willingness to participate in PPPs.

In sum, these financial trade-offs created an economic misalignment, where the incentive structures of PPPs failed to adequately compensate firms for opportunity costs associated with global equity objectives.

(c) Distribution constraints

There is a significant disparity in vaccine delivery infrastructure across different regions. Although mRNA vaccines were widely used during the pandemic, their stringent cold-chain requirements posed substantial challenges to equitable distribution. Given that international PPP initiatives, such as COVAX, primarily address vaccination inequalities and support LMICs with limited infrastructure, mRNA vaccine manufacturers must evaluate the feasibility and importance of participating in PPPs to mitigate delivery risks. Moreover, effective global vaccine distribution requires advanced storage facilities and skilled personnel—resources that are typically concentrated in high-income countries (34). Companies must also consider product and market suitability when making strategic decisions.

In conclusion, these infrastructural disparities reflected a structural misalignment between global PPP objectives and the material capacities required for equitable vaccine delivery.

(d) Contractual obligations

Government financial support to private firms is typically provided through the signing of pre-purchase agreements. The contractual obligations are closely linked to public funding and significantly influence the firms’ capacity to engage in PPPs. Some open-access contracts demonstrate that nations have preferential access to vaccine doses (22), which initially restricts firms’ participation in COVAX. For instance, the contract between the United Kingdom and AstraZeneca includes a clause stating that if AstraZeneca or its subcontractors are compelled to delay the supply of vaccine doses, the government has the right to terminate the agreement (35). These binding commitments, coupled with limited production capacity, forced companies to prioritize bilateral contracts over global partnerships, even when they were otherwise supportive of PPP goals.

Therefore, these contractual dependencies generated an institutional misalignment, where public procurement frameworks unintentionally undermined the collaborative flexibility required for global PPP operations.

#### 3.3.3 Drivers for public sector participation in the PPP

The public sector primarily includes governmental agencies, national health authorities, and public research institutions. Governments adopt PPPs as strategic instruments of health governance to mitigate pandemic risks while balancing domestic and international obligations. Most studies discuss these motivations in the context of COVAX, which provides a useful lens for understanding public-sector engagement.

(a) Enhancing national image and global standing

Governments often view PPP participation as a method to demonstrate international solidarity and ethical leadership. Through COVAX, many high-income countries (HICs) contributed funds and vaccines, presenting themselves as key global health actors (7, 36). Despite these efforts, there has been criticism directed at some wealthy nations for hoarding vaccines, although COVAX has facilitated their transition into charitable donors (25). From an ethical and human rights perspective, vaccines are public goods essential to fulfilling states’ obligations to protect health for all (37). Wealthy countries are expected to respect, protect, and assist lower-income populations through practical mechanisms such as COVAX. This engagement therefore serves as both a normative commitment and a diplomatic strategy to enhance reputational legitimacy. Norway exemplifies this logic through its consistent support for multilateral vaccine access initiatives.

(b) Protecting national interests

Beyond ethical imperatives, governments also engage in PPPs to safeguard domestic interests under fiscal and political constraints. For low- and middle-income countries (LMICs), PPP participation secures affordable access to vaccines through donor-funded mechanisms such as the Gavi COVAX AMC (33). As less powerful partners, their motivation lies in ensuring domestic supply without excessive fiscal burden. Several studies delve into the reasons why HICs are interested in PPPs. Expert interviews reveal that upper-middle-income countries aim to gain access to vaccines for up to 20% of their population through COVAX, primarily because COVAX can provide a diverse portfolio of vaccines (36). Additionally, COVAX employed a fair allocation model, ensuring that all participating countries receive vaccines, irrespective of their income level or the urgency of their situation (8). However, COVAX does not prohibit bilateral agreements between participating countries and manufacturers (30). This additional option allows wealthier countries to stockpile vaccines, often placing them in a more advantageous position due to the limited availability of vaccines and their nature as a commodity. Lexchin (18) illustrates this point by citing Canada’s acquisition of 1.9 million doses of the Astra-Zeneca vaccine at a time when many African countries and other LMICs had yet to receive any doses.

PPPs also advance national security objectives. The United States, for instance, procured 1 billion Pfizer doses through COVAX at cost, with 70% earmarked for donation. Under the Defense Production Act, these doses could be redirected for domestic use if needed—an approach described as proxy procurement (25). Such arrangements enhanced both global reputation and domestic preparedness. Over time, PPP institutionalization may also reinforce local vaccine manufacturing and reduce future dependence on imports, as demonstrated by the UK Vaccine Task Force (38).

(c) Institutional incentives for public engagement

Global PPPs such as the Access to COVID-19 Tools Accelerator (ACT-A) illustrate how public institutions view partnership models as efficient governance tools. The initiative’s COVAX Facility became the largest and best-funded platform within ACT-A, pooling resources and sharing R&D risks among multiple actors (34). Scholars argue that PPP frameworks enabled governments to balance national vaccine nationalism with global equity objectives by mediating between pragmatic and cosmopolitan policy logics (36).

COVAX thus represented a historic effort to establish a collective allocation mechanism under international coordination (7). Its inclusive governance structure allowed governments to pursue public-value objectives while retaining flexibility for domestic accountability. Although its implementation fell short of expectations, PPPs remain a pragmatic institutional pathway for governments to align global responsibility with national interest.

Overall, this section clearly shows that governments engage in PPPs for normative, strategic, and institutional reasons, linking ethical responsibility with national interest and governance efficiency.

#### 3.3.4 Barriers to public sector participation in the PPP

Despite the strategic and ethical motivations discussed earlier, several factors have constrained the public sector’s willingness and capacity to participate in PPPs during the COVID-19 pandemic. The identified barriers can be grouped into two main domains: political nationalism and institutional inertia, both of which contributed to governments’ limited engagement in global partnership mechanisms.

(d) Vaccine nationalism

Many HICs endowed with substantial financial resources and vaccine production capabilities insist on prioritizing their own populations to varying extents (7). Consequently, these nations opt for bilateral agreements and vaccine stockpiling rather than contributing to or engaging in global PPP initiatives. In the most extreme cases, such HICs exhibit an unwillingness to assume any responsibility for individuals residing beyond their national borders (39), an attitude that often remains implicit among these countries. The discourse surrounding the commodification of vaccines frequently centers on their dual nature as both competitive and exclusive goods, aligning them with market trade principles (40). Due to the finite supply of vaccines in the global market, COVAX and similar partnerships face competition from governmental entities when procuring vaccines. This undermines the leadership of COVAX in equitable resource allocation (8). While numerous scholars argue that vaccines should be classified as global public goods to ensure global health security, persuading vaccine manufacturers to relinquish intellectual property rights and share profits proves challenging. Despite COVAX and key organizations advocating for intellectual property waivers, certain public entities remain hesitant and resistant, as evidenced by their actions. Even under some circumstances, some donor countries resorted to donations via bilateral country agreement as a tool of soft power diplomacy to protect their own interests.

(e) Operational constraints of PPP model

Beyond political considerations, institutional inertia and administrative fragmentation, reflected in slow decision-making and weak inter-agency coordination also limited governments’ participation in PPPs. Some governments opted for direct procurement from manufacturers, perceiving PPP mechanisms as too slow or complex to meet urgent needs. The success of PPPs often depends on early governmental coordination, yet the pandemic underscored the critical importance of speed and administrative agility.

For instance, institutional inertia was evident in COVAX’s delayed procurement process, where multi-level approval chains postponed contract signing by 4 months (30). Similarly, administrative fragmentation emerged in fragmented data systems and overlapping jurisdictions, as illustrated by the United States’ Federal Retail Pharmacy Program (FRPP), which struggled with vaccine-data sharing across federal and state levels (41).

In sum, the bureaucratic rigidity and fragmented authority discouraged governments from engaging in PPPs, despite the availability of political will and global incentives.

## 4 Discussion

This study analyses the drivers and barriers that influence the public and private sectors’ decisions to engage in PPPs within the COVID-19 vaccine supply chain. Drawing on 21 peer-reviewed studies, we introduce and apply an author-developed Governance Alignment Framework (GAF) that conceptualizes these factors as manifestations of governance coherence (42). The GAF spans levels of governance (global, regional, and national) and supply-chain stages (R&D, regulatory authorization, procurement, distribution, and vaccination/administration). The time window for evidence synthesis is Dec 2019-Oct 2025.

Governments joined PPPs to secure supply, signal global credibility, and balance national and international obligations. Firms participated to obtain financial stability, regulatory facilitation, and reputational legitimacy. Yet both sectors faced obstacles such as political nationalism, market competition, contractual rigidity, and administrative fragmentation. Governments and firms were motivated by different but interdependent considerations.

Rather than evaluating performance, this study focuses on how governance mechanisms align or separate these incentives. When institutional arrangements such as pooled procurement, indemnification clauses, or regulatory coordination enable mutual responsiveness between public and private actors, participation becomes more coherent. When internal contradictions within each actor disrupt that relationship, alignment weakens.

Within this framework, institutional mechanisms denote the formal and informal arrangements that enable cooperation between public and private actors across financial, regulatory, legal, and administrative domains. These arrangements include instruments such as advance market commitments, risk-sharing and indemnification clauses, harmonized regulatory approvals, and multilateral coordination platforms such as the Federal Retail Pharmacy Program (FRPP) and COVAX. Through these mechanisms, PPPs establish the rules of engagement by defining how resources, responsibilities, and risks are allocated and governed across partnerships. The effectiveness of these mechanisms determines whether public and private partners succeed in translating shared goals into coordinated action through mutual incentives, accountability structures, and trust-building processes, or remain fragmented by power asymmetries and conflicting interests. Ultimately, alignment represents the core condition of governance coherence, serving as the functional link between sectoral motivations and governance outcomes.

Based on this framework, this study integrates Public Value (11), Resource-Dependence (12), Legitimacy/Stakeholder (13), and Public Goods theories (14) within the governance alignment framework to provide a multi-lens for analyzing the governance dynamics of PPPs in the COVID-19 vaccine supply chain. (1) From a Public value perspective, governments participate in PPPs to protect collective profits to ensure the health equity and national security, seeking to translate private resources into public outcomes. (2) Resource-Dependence Theory explains the collaboration requirements for stakeholders. Public authorities depend on private innovation, research capacity and logistics, while private firms rely on public funding, regulation and legitimacy under the crisis. (3) Stakeholder and Legitimacy Theories emphasize why both sides are pursuing reputational and general approval that governments need to demonstrate the responsible national image, and enterprises need to fulfill the CSR and maintain market trust. Finally, (4) Public Goods Theory illustrates the rationale for PPP formation within COVID-19 vaccine supply chain. Vaccines, as global public goods, require cross-sectoral coordination to overcome market failure and prevent the issue of distribution inequity. These four theoretical perspectives clarify together how institutional mechanisms determine the alignment between public and private sectors.

### 4.1 The governance alignment framework

**Figure 2:**
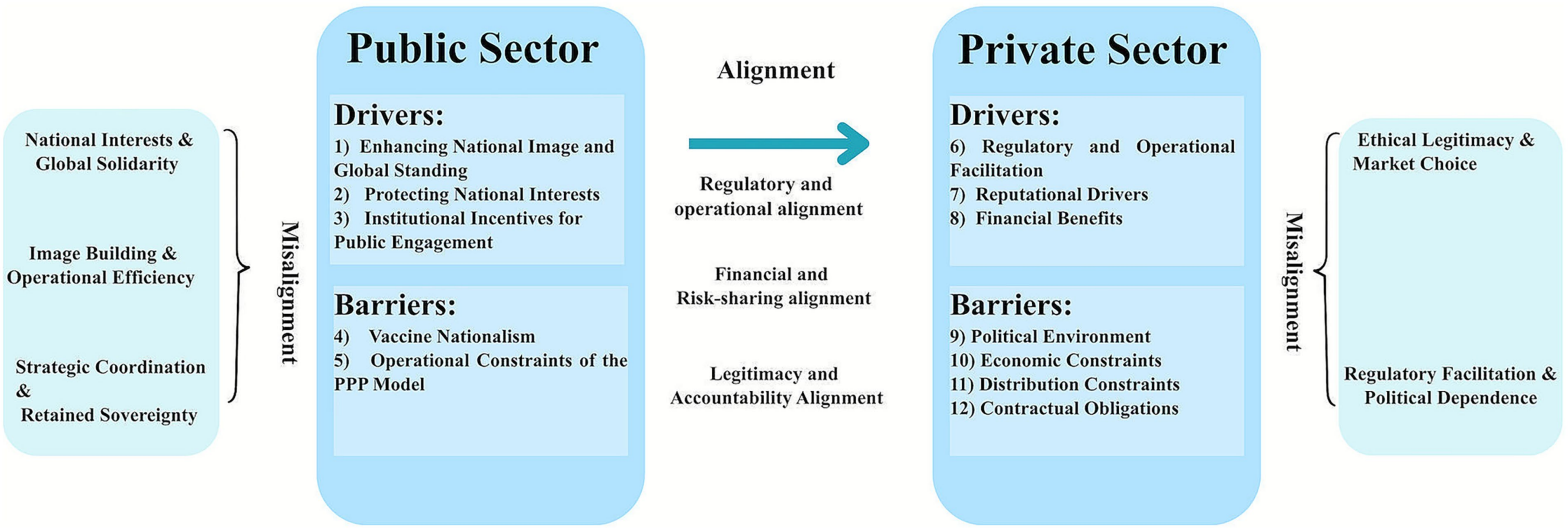
Governance alignment and misalignment in PPPs for COVID-19 vaccine supply. Left panel: public sector; right panel: private sector; center arrow: cross-sector alignments. Each panel lists sector-specific drivers and barriers; outer brackets denote within-sector misalignment themes. Numbered keys map to the Results text: (1–3) = 2.3.3 (A–C); (4–5) = 2.3.4 (A,B); (6–8) = 2.3.1 (A–C); (9–12) = 2.3.2 (A–D). PPP, Public–private partnership.

The Governance Alignment Framework explains the formation of PPPs through the relationships among drivers and barriers that influence public and private sectors’ desire. It examines whether and how public motivations influence private responses, and how internal tensions within each sector affect that influence. The framework treats alignment and misalignment as a way of interaction, not as performance outcomes. Figure 2 shows the interaction and mechanism of this framework.

Alignment indicates a consistent connection between public and private incentives. When political commitment, fiscal support, and regulatory support from governments encourage private companies’ engagement, the relationship is aligned.

Misalignment always arises from the internal contradictions within the sector, leading to an inconsistent relationship among these motivations.

### 4.2 Misaligned governance relationships

#### 4.2.1 Misaligned governance relationships within the public sector

The findings presented in Table 1 reveal that there are horizontal, misaligned relationships among internal factors for the public sectors. From a governance alignment perspective, these horizontal tensions indicate that institutional mechanisms have not fully harmonized public motivations toward cooperation by participating in PPP. Based on four theories referred, we found three main distinct misalignments.

**Table 1:** Public sector misalignments.

Conflict theme	Driver	Barrier	Governance interpretation
National interests and global solidarity	Protecting national interests. Promoting global solidarity	Vaccine nationalism	Reflects a misaligned relationship between national protectionism and global equity. Based on the public goods theory, national self-interest weakens global cooperation and PPP legitimacy.
Image building and operational efficiency	Enhancing national image and global standing	Operational constraints (institutional inertia and administrative fragmentation)	Reveals the conflict between stakeholder driven symbolic participation and the slow visibility of PPP. It reduces the alignment between ethical aspirations of public sectors and institutional capacity.
Strategic coordination and retained sovereignty	Institutional incentives for public engagement (pooling, coordination, funding, risk sharing)	Vaccine nationalism	Shows how recognition of the PPP model coexists with asymmetric resource dependence and state reluctance to cede control to multilateral mechanisms.

The first one is the misalignment between the protection of national interests and the promotion of global solidarity. Although governments recognized the collective value of global vaccine distribution, their actions often reflected the pursuit of self-interest (14) described by Public Goods Theory. As mentioned above, both purchasing vaccines through PPPs and bilateral deals stem from vaccine nationalism, with the objective of protecting national interests. However, these two approaches yield different outcomes. In practice, most wealthy nations opt for bilateral deals with private companies and use COVAX doses as a supplementary measure. The mechanism to mitigate this conflict is the COVAX purchase arrangement, which allows participating countries to choose between a Committed Purchase Arrangement or an Optional Purchase Arrangement (43). COVAX does not impose any coercive measures to prevent bilateral agreements but instead relies on appeals and encouragement. Health officials have noted that these bilateral deals risk undermining equitable global vaccine distribution because many individual agreements may ignore vulnerable groups (44). It can be argued that this contradiction is resolved by PPPs sacrificing some of their own competitiveness to attract countries with significant influence in the international vaccine market. Finally, COVAX resorted to a traditional aid-financing approach, placing affluent governments and profit-driven corporations in control over lower-income countries (45). Several researchers attribute the failure of global PPPs to the dominant position of rich countries and the weakness of global health organization (9), which can also be attributed to compromises resulting from internal national conflicts.

The second misalignment appears between the image building and operational efficiency. Rich governments often joined PPPs in COVID-19 vaccine supply chain to express their solidarity and credible image and seek reputational approval from the global audiences, which can be explained by Stakeholder and Legitimacy Theories. However, the slow and bureaucratic procedures characteristic of multilateral PPPs, such as COVAX harm the pursuit of operational efficiency for governments, and this reflects the erosion of public-value coherence.

The last alignment is from the coexistence of strategic coordination and retained sovereignty. Governments admit the advantage of PPP model in vaccine source pooling, risk sharing and financial benefits, but remain reluctant to give up the control over vaccine procurement and distribution and wealthier countries maintained high influence over governance decisions in the PPPs. The logic of Resource-Dependence Theory support this idea because it highlights the preservation of dominance over critical resources when seeking cooperation (12).

In summary, these horizontal, misaligned relationships within public sector reveal how competing governance rationales coexist and undermine governance coherence. Viewed through the Governance Alignment Framework, these findings illustrate that governance coherence depends on the effectiveness of institutional mechanisms in translating public value objectives into practical actions. When it fails, the alignment will be broken, and hybrid governance arrangements lose their integrative function in this process.

#### 4.2.2 Misaligned governance relationships within the private sector

The findings presented in Table 2 show that misaligned relationships within the private sectors are conflicts between legitimacy-seeking and market-driven motivations. From our governance theoretical view, two forms of misalignment are summarized.

**Table 2:** Private sector misalignments.

Conflict theme	Driver	Barrier	Governance Interpretation
Regulatory facilitation and political dependence	Regulatory and operational facilitation	Political environment influence	Reveals a misaligned relationship between firms’ request of operational efficiency and their dependence on politicized governance contexts.
Ethical legitimacy and market choice	Reputational drivers	Economic constraints (Profit Orientation and Market Competition)	Reflects the conflict between ethical legitimacy and commercial pragmatism.

The first one is the conflict between regulatory facilitation and political dependence. While participating in PPPs, private pharmaceutical firms gain regulatory streamlining in vaccine approval, logistical support in vaccine distribution, and legal protection, such as indemnification clauses. However, these benefits were determined by political interference and vaccine diplomacy. Companies’ performance was constrained by governments’ national strategies and geopolitical competitions, resulting in delayed cooperation and late access to the market. Firms rely on public funding, authorizations and political legitimacy, but such dependency limits horizontal alignment between public objectives and private pursuit.

Another ambivalence in corporate decision-making lies in the tension between ethical legitimacy and market choice. There is tension across all sectors, but the pandemic emergency and the extreme imbalance between supply and demand in the market have exacerbated this conflict between ethical benefits and market profits. Based on equilibrium and stakeholder theories (46), the pandemic has exposed the vulnerability of companies to external forces, compelling them to prioritize immediate profits for survival at the expense of social responsibility funding (47). Even pharmaceutical companies face pressure regarding vaccine development. A fundamental question arises: Can businesses effectively balance their financial goals with their social responsibilities? For pharmaceutical companies, collaborating with COVAX represents a key approach to fulfilling their corporate political social responsibility by ensuring equitable access to COVID-19 vaccines (48). The largest PPP in the COVID-19 vaccine distribution employed various strategies to ensure market profits and attract enterprises, which aligns with the PPP cooperation model. However, examples cited by De Bengy Puyvallée and Storeng (25) highlight that some pharmaceutical companies prioritized market profits over social responsibility, indicating that PPP concessions did not lead to significant positive feedback. Researchers have identified several issues arising from this conflict, including inequitable benefits for recipient countries (8), difficulties in technology sharing (30), differences in vaccine pricing (29), and non-transparent purchase contracts (26). This conflict demonstrates that while PPPs can generate reputational incentives for private sectors, they struggle to sustain public value (11) when economic gains dominate ethical obligations. Therefore, balancing PPP success with company profits should be a critical focus moving forward. An interesting phenomenon is the overlooked role of third parties, especially international health organizations. Ideally, they should serve as coordinators but have been marginalized due to a lack of influence.

In sum, the governance misalignment illustrates the structural tension in the model of COVID-19 vaccine PPPs, and the commercial logics and public responsibility are coexisting together in a fragile hybrid governance system.

### 4.3 Aligned governance relationships between public and private sectors

The aligned governance relationship emerges primarily along a vertical dimension between public and private sectors, which is realized through institutional mechanisms that combine the national power and market capacities. Even if there are some barriers continue to influence the degree of coherence, the governance alignment makes an effect when regulatory flexibility, financial incentives and ethical request are integrated across public and private sectors. There are three aligned relationships summarized as below (Table 3):

**Table 3:** Cross-sector alignments.

Coherence theme	Public sector	Private sector	Governance interpretation
Regulatory and operational alignment	Drivers: Protecting national interests Barriers: Vaccine nationalism	Drivers: Regulatory and operational facilitation Barriers: Political Environment Influence	Reveals partial alignment between governmental regulatory and private efficiency needs.
Financial and risk-sharing alignment	Drivers: Institutional incentives for public sector Barriers: Vaccine nationalism	Drivers: Financial Benefits Barriers: Economic Constraints	Shows how risk-sharing and financial incentives facilitate joint investment and collective benefit creation within PPPs.
Legitimacy and Accountability Alignment	Drivers: Enhancing National Image and Global Standing Barriers: Vaccine nationalism	Drivers: Reputational drivers Barriers: Contractual obligations	Demonstrates the alignment between ethical legitimacy and shared accountability.

The first one is regulatory and operational alignment. The public sector’s regulatory authorities and the private sector’s operational efficiency goals manage to work together, which reflect in the vaccine research and development. For example, the government frequently provides administrative support to private companies. Administrative support primarily manifests in expediting the research and approval processes for vaccine candidates. The key organizations involved in vaccine approval include the International Coalition of Medicines Regulatory Authorities (ICMRA), the European Medicines Agency (EMA), and the U. S. Food and Drug Administration (FDA). Notable private sector representatives include the International Federation of Pharmaceutical Manufacturers and Associations (IFPMA). Under the framework of ICMRA, the longstanding partnership between the EMA and FDA was further strengthened by the challenges posed by COVID-19, exemplified by their joint discussions on pre-clinical data requirements and potential regulatory harmonization. Priority in the approval process involves close collaboration between the FDA and applicants, as well as other agencies, to expedite the quality assessments of vaccines (48). Additionally, the EMA has been instrumental in expanding manufacturing sites for enterprises to enhance production capacity and supply of COVID-19 vaccines in the EU.

Then, the financial and risk-sharing alignment represents the core of the PPP governance model. Instruments such as advance market commitments, push-pull funding, and COVAX redistributed investment risk and incentivized vaccine production for collective global benefit followed the principles of Public Goods Theory (14). However, this alignment was partial and conditional. Vaccine nationalism, adopted by various nations, can significantly impact the political environment and create market challenges. From the outset of vaccine research and development, competition among superpowers such as the US, China, and Russia may hinder the successful development and deployment of vaccines. During the phases of vaccine procurement and distribution, vaccine nationalism is primarily manifested through bilateral deals promoted by governments rather than through PPPs. These bilateral agreements could negatively affect pricing and availability, as wealthier countries might outbid others for limited vaccine supplies (49), leading to greater control over pricing by enterprises. Consequently, vaccine nationalism undermined the potential of vaccines as a global public good.

The last one is the legitimacy and accountability alignment, demonstrates how the ethical responsibilities were translated to the institutional mechanism in the practice. For example, Governments participate in COVAX to demonstrate ethical leadership and moral responsibility, while private sectors aim to reinforce their corporate legitimacy by contributing to equitable vaccine access. From a public value perspective, this process aims to embed ethical intent to operational design.

### 4.4 Theoretical integration

We link the evidence to the four theories as follows. Regulatory-operational alignment is most consistent with Resource Dependence theory and Public Value theory. The joint and interoperable data, and logistic are used to reduce uncertainty. Speed and safety are public outcomes. Then, the process of vaccine approval represents the Legitimacy/Stakeholder theory. Financial and risk-sharing alignment combines Resource-Dependence (APAs, indemnity and pre-funding stabilize production) with the public-goods rationale (pooling under scarcity), again underwritten by public value. Legitimacy–accountability alignment is driven by Legitimacy/Stakeholder expectations and enacted through Public-Value procedures (disclosure, allocation rules, audit trails), often unlocking resources in Resource-Dependence terms. On this basis, the GAF is a descriptive-analytical framework: it organizes reported configurations (drivers, barriers, mechanisms, alignment/misalignment) and does not make predictive claims or performance ratings. The three alignment types used here are analytic categories that can be refined in future applications.

However, viewed through collaborative governance, the power asymmetries in our corpus operate as adverse starting conditions and process biases: when critical resources, decision rights, or agenda-setting capacity are concentrated at a few nodes, principled engagement is tilted, shared motivation is fragile, and capacity for joint action remains under-specified (50, 51). In our GAF, this maps directly onto misalignment. For example, on the public side, national interest vs. global solidarity and image vs. operations reflect uneven authority and competing accountability; on the private side, ethical legitimacy vs. market choice reflect dependence on public policies and geopolitics (52). Alignment becomes more likely when design features buffer asymmetry. We therefore interpret misalignment as a collaborative-governance shortfall rooted in unequal power structures, rather than a performance verdict.

### 4.5 Limitations and risks of biases

This study acknowledges limitations that also point to avenues for future work. First, scope and unit of analysis. Distinguishing COVAX from other PPPs in the COVID-19 vaccine supply chain is non-trivial. Although COVAX was the largest PPP of the pandemic, it did not span all supply-chain stages and operated in practice as an umbrella bundling multiple institutional arrangements. Its prominence in the literature is typical of crisis governance and publication dynamics.

Additionally, PPPs involved in the vaccination phases have been largely overlooked. The traditional vaccine supply chain comprises several stages, including research, approval, procurement, distribution, and administration. This means our analysis most strongly characterize global and downstream phases. Transfer to purely domestic or bilateral PPPs or non-crisis periods should be cautious, where the liability regimes, pricing formulas, and documentation practices differ.

Second, this research lacks an introduction to the roles of third parties. Both international organizations and non-governmental organizations (NGOs), such as the Bill & Melinda Gates Foundation, are important participants in PPPs. Their relationships with the public sector warrant discussion. One possible reason for their limited inclusion may be the relatively weak voice and marginalization of these organizations. Nevertheless, they undoubtedly play crucial roles in global health PPPs. The lack means analyses may over focus on the state influence on the companies and the under-state mediation effects.

Mentioning the quality of studies and potential risks of bias, across the 21 articles, most contributions are narrative or qualitative policy analyses, with relatively few interview-based or data-driven designs. Recurrent strengths include clear case descriptions, traceable documentation, and explicit normative premises. Recurrent limitations include HIC/English emphasis, reliance on official or multilateral documentation, and limited disclosure of sampling frames. We therefore refrain from frequency claims and treat counts as indicators of reporting salience; qualitative conclusions are framed as patterns in the published record, not prevalence estimates.

## 5 Conclusion

This systematic review proposed a Governance Alignment Framework (GAF) to analyze the relationships among factors that influence public and private sectors’ decisions to participate in PPPs. Viewed through the lenses of Public Value, Resource Dependence, Legitimacy or Stakeholder, and Public Goods, these relationships clarify how normative aims, interdependence, legitimation needs, and collective action constraints coexist in a fragile hybrid governance system.

Achieving alignment among factors is essential not only for mobilizing resources but also for ensuring the resilience and equity of vaccine supply chain. Because the evidence base skews toward global or HIC settings and distribution-stage artifacts, future work should broaden LMIC sources and apply lightweight text mining and network mapping to enable comparisons within different economies.

## Data Availability

The original contributions presented in the study are included in the article/Supplementary material, further inquiries can be directed to the corresponding author/s.
