# Supplementary material for: A systematic review of global COVID-19 vaccine PPPs: drivers and barriers to governance alignment
Source: Front Public Health. 2025 Dec 1;13:1727808. doi: 10.3389/fpubh.2025.1727808 (PMC12706164; doi:10.3389/fpubh.2025.1727808)
Supplement: Supplementary file 1 [file Table_1.docx]

Appendix 1

Table S1. WoS Search Configuration Overview

| Item | Content |
| --- | --- |
| Database | Web of Science Core Collection |
| Research Period | 1 Jan 2020 – 1 Sep 2025 |
| Search Field | Topic (TS: Title/Abstract/Author Keywords/Keywords Plus) |
| Search Query (Topic) | ("Public-Private Partnership" OR "PPP" OR "Private Finance Initiative" OR "PFI" OR "Public-Private Cooperation" OR "PPP* Collaboration" OR "Inter-organizational" OR "Public-Private Relationship" OR "Public Enterprise*" OR "Public Alliance*" OR "Non-profit Partnership*" OR "Private Sector Contracting" OR "COVAX" OR "Operation Warp Speed" OR "OWS") AND ("COVID-19 Vaccine" OR "COVID Vaccine*" OR "Coronavirus Vaccine*") |
| Document Type | Article |
| Language | English |
| Research Areas | Public, Environmental & Occupational Health; Infectious Diseases; Health Care Sciences & Services; International Relations; Immunology; Medical Ethics; Experimental Medicine; Social Issues; Business & Economics; Public Administration; Government & Law; Area Studies; Social Sciences (Other Topics); Environmental Sciences & Ecology; Microbiology; Engineering; Legal Medicine; Operations Research & Management Science; Pharmacology & Pharmacy; General & Internal Medicine; Allergy; Biotechnology & Applied Microbiology; History; Philosophy; Sociology; Tropical Medicine; Virology; Biomedical Social Sciences. |

Note: In WoS, Topic (TS) searches Title/Abstract/Author Keywords/Keywords Plus.

| Table S2. Checklist tool for eligibility criteria (n= 99) |  |  |  |
| --- | --- | --- | --- |
| **Selection Criterion** | **Yes** | **No** | **Unclear** |
| The study examined PPPs within the context of the COVID-19 vaccine supply chain. | 60 | 35 | 4 |
| An empirical or descriptive approach was utilized to examine PPPs implementation in the COVID-19 vaccine supply chain. | 21 | 76 | 2 |
| Discussion regarding drivers and barriers of stakeholders’ participation in PPPs was provided. | 28 | 37 | 34 |
| The PPPs did not concern the health status of a specific demographic, and deliberations were conducted at least at the national or multinational level. | 62 | 37 | 0 |
| **Overall appraisal** |  |  |  |
| Include | 21 | n/a | n/a |
| Exclude | 78 | n/a | n/a |
