# Supplementary material for: A systematic review of global COVID-19 vaccine PPPs: drivers and barriers to governance alignment
Source: Front Public Health. 2025 Dec 1;13:1727808. doi: 10.3389/fpubh.2025.1727808 (PMC12706164; doi:10.3389/fpubh.2025.1727808)
Supplement: Supplementary file 2 [file Table_2.docx]

| No | Source | PPP case | Targeted Area | Level |
| --- | --- | --- | --- | --- |
| 1 | (Manriquez Roa et al., 2021) | COVAX | World | Global |
| 2 | (Eccleston-Turner & Upton, 2021) | COVAX | World | Global |
| 3 | (Sung et al., 2021) | COVAX | World | Global |
| 4 | (Pilkington et al., 2022) | COVAX | World | Global |
| 5 | (Florio, 2022) | No single case | US | National |
| 6 | (Fajber, 2022) | COVAX | World | Global |
| 7 | (De Bengy Puyvallée & Storeng, 2022) | COVAX | World | Global |
| 8 | (Choi et al., 2023) | No single case | US | National |
| 9 | (Kana et al., 2023) | No single case | Africa | Regional |
| 10 | (von Achenbach, 2023) | COVAX | World | Global |
| 11 | (Kim et al., 2023) | FRPP | US | National |
| 12 | (Forman et al., 2023) | COVAX | World | Global |
| 13 | (Lexchin, 2023) | No single case | Canada | National |
| 14 | (Holzer et al., 2023) | COVAX | World | Global |
| 15 | (Storeng et al., 2023) | COVAX | World | Global |
| 16 | (Carroll et al., 2024) | VTF | UK | National |
| 17 | (Scholz et al., 2024) | COVAX | World | Global |
| 18 | (Jones-Jack et al., 2024) | FRPP | US | National |
| 19 | (O’Hara et al., 2024) | COVAX | World | Global |
| 20 | (Wolff & Ladi, 2025) | COVAX | EU | Regional |
| 21 | (Fawole et al., 2025) | COVAX | World | Global |

Appendix 2. List of Literature included.
